# A conjoint analysis of bulk RNA-seq and single-nucleus RNA-seq for revealing the role of ferroptosis and iron metabolism in ALS

**DOI:** 10.3389/fnins.2023.1113216

**Published:** 2023-03-02

**Authors:** Xiujuan Fu, Yizi He, Yongzhi Xie, Zuneng Lu

**Affiliations:** ^1^Department of Neurology, Renmin Hospital of Wuhan University, Wuhan, China; ^2^Department of Lymphoma and Hematology, Hunan Cancer Hospital, The Affiliated Cancer Hospital of Xiangya School of Medicine, Central South University, Changsha, China; ^3^Department of Radiology, The Third Xiangya Hospital, Central South University, Changsha, China

**Keywords:** amyotrophic lateral sclerosis, ferroptosis, iron metabolism, single nuclear analysis, WGCNA, LASSO

## Abstract

Amyotrophic lateral sclerosis (ALS) is a neurodegenerative disease characterized by progressive and selective degeneration of motor neurons in the motor cortex of brain and spinal cord. Ferroptosis is a newly discovered form of cell death and reported to mediate selective motor neuron death in the mouse model of ALS. The growing awareness of ferroptosis and iron metabolism dysfunction in ALS prompted us to investigate the expression pattern of ferroptosis and iron metabolism-related genes (FIRGs) in ALS. Here, we performed a conjoint analysis of bulk-RNA sequence and single-nucleus RNA sequence data using the datasets from Gene Expression Omnibus (GEO) to reveal the role of FIRGs in ALS, especially in selective motor neuron death of ALS. We first investigated the differentially expressed genes (DEGs) between ALS and non-neurological controls. Weighted gene co-expression network analysis constructed the gene co-expression network and identified three modules closely associated with ALS. Fifteen FIRGs was identified as target genes based on least absolute shrinkage and selection operator regression analysis as follows: ACSL4, ANO6, ATP6V0E1, B2M, CD44, CHMP5, CYBB, CYBRD1, HIF1A, MOSPD1, NCF2, SDCBP, STEAP2, TMEM14C, ULK1. These genes could differentiate ALS patients from non-neurological controls (*p* < 2.2e−16) and had a valid value in predicting and diagnosing ALS (AUC = 0.881 in primary dataset and AUC = 0.768 in validation dataset). Then we performed the functional enrichment analysis of DEGs between ALS cases, the most significantly influenced by target genes, and non-neurological controls. The result indicated that the most significantly influenced functions in ALS pathogenesis by these identified FIRGs are synapse pathways, calcium signaling pathway, cAMP signaling pathway, and phagosome and several immune pathways. At last, the analysis of single- nuclear seq found that CHMP5, one of the 15 FIRGs identified by bulk single-nucleus RNA-seq data, was expressed significantly higher in ALS than pathologically normal (PN), specifically in excitatory neuron populations with layer 2 and layer 3 markers (Ex L2_L3), layer 3 and layer 5 markers (Ex L3_L5). Taken together, our study indicates the positive correlation between FIRGs and ALS, presents potential markers for ALS diagnosis and provides new research directions of CHMP5 function in selective motor neuron death in ALS.

## Introduction

Amyotrophic lateral sclerosis (ALS) is a severe neurodegenerative disease characterized by progressive and relatively selective loss of upper and lower motor neurons. It was brought into the spotlight due to its lethality and complexity. However, foggy pathogenesis and uncontrolled progression still confuse the world.

Growing evidences indicate that iron affects cell death pathways and brain homeostasis in neurodegenerative diseases. Ferroptosis is a type of cell death triggered by the built up of lipid peroxides, defined as an iron-dependent regulated necrosis that can affect neurons (Stockwell et al., 2017). Since iron is a redox ion, it can induce free radicals, including reactive oxygen species (ROS), which participates in the regulation of cell survival and death. ROS can cause cell damage by destroying the iron homeostasis of cells, leading to a vicious circle (Sirabella et al., 2018). Evidence from past decades links iron homeostasis to neuronal cell death in ALS (Matsuo et al., 2021). In ALS patients, MRI reveals increased iron accumulation in motor cortex (Kwan et al., 2012). The markers of lipid oxidation and iron status are correlated with clinical function decline in ALS (Devos et al., 2019). Another study showed that moderate iron chelation regimen may incorporate a novel curative option for neuroprotection in ALS (Moreau et al., 2018). ALS is the most common motor neuron disorder, and show selective vulnerability of motor neurons in cortex (Hammer et al., 1979; Cronin et al., 2007). Excitingly, the research in utilizing ALS SOD1^G93A^ mice model showed that ferroptosis could mediate selective motor neuron death (Wang et al., 2022). Focus on ferroptosis and iron metabolism in ALS may provide new targets to uncover the pathogenesis of selective vulnerability of motor neurons and reduce dysfunction.

Here we did a conjoint analysis of bulk RNA-seq and single-nucleus RNA-seq based on the publicly available mRNA expression data. In bulk RNA-seq analysis, we focused on differentially expressed genes and altered pathways related to ferroptosis and iron metabolism in ALS to uncover the involvement of ferroptosis and iron metabolism in ALS and provide potential targets for further investigation. In single-nucleus RNA-seq analysis, we explored the expression of hub genes in primary cortex motor neurons in ALS to explore the role of ferroptosis and iron metabolism in motor neurons and provide potential pathogenesis of selective vulnerability of motor neurons in ALS.

## Materials and methods

### Data preparation for bulk RNA-seq

Transcriptome data were obtained from Gene Expression Omnibus (GEO). Specifically, the bulk RNA-seq dataset is from GSE153960 (Prudencio et al., 2020), from which we extracted two parts of the data. A total of 684 ALS spectrum motor neuron disease (MND) patients and 190 non-neurological controls were selected in the primary dataset for hub genes screening, grounded on the platform of GPL24676. Another dataset based on the platform of GPL16791 including 546 ALS spectrum MND patients and 90 non-neurological controls for validation. The raw count matrices were normalized and transformed into fragments per kilobase of sequence per million mapped reads (FPKM) values for further analysis.

### Screening of differentially expressed genes (DEGs)

The raw counts of expression profiles were extracted from the primary dataset (GPL24676), generating the heatmap using the R package “pheatmap.” The DEGs were screened using the DESeq2 R package with a threshold of | log2FC| ≥ 0.5 and FDR < 0.05 (Love et al., 2014). A volcano plot was used to visualize the distribution of DEGs.

### Ferroptosis and iron metabolism-related genes screening and analysis

The ferroptosis-related genes were downloaded from FerrDb database (FerrDb)^1^ (Zhou and Bao, 2020; Zhou et al., 2022). A total of thirty-two iron metabolism-related gene sets were extracted from the Molecular Signatures Database (MsigDB)^2^ and Gene Ontology with keywords “iron” and “heme.” We combined the ferroptosis-related genes and iron metabolism-related genes and removed the repeated genes. Finally, a total of 763 ferroptosis and iron metabolism-related genes (FIRGs) were screened out (as shown in the Supplementary Table 1).

### WGCNA

Weighted gene co-expression network analysis (WGCNA) was performed on the DEGs extracted from the primary dataset, based on the WCGNA analysis in the R package (Zhang and Horvath, 2005; Li et al., 2020). The log2 transformed gene expression data was used to calculate the Pearson correlation matrix. We used R function “pickSoftThreshold” for module building and screened the soft threshold power β to achieve a scale-free topology. The minimum number of genes per gene module was set to 30, and genes were grouped into modules with similar expression patterns. Finally, we identified modules associated with ALS by assessing correlations between phenotypes and modules by Pearson correlation. Besides, a WGCNA integrated function (module Preservation) was applied to calculate module preservation statistics. Zsummary was used to assess the significance of observed statistics by distinguishing preserved from non-preserved modules *via* permutation testing 200 times (Langfelder et al., 2011). Preservation analysis was performed in primary dataset (GPL24676) as 1:1 divided into training set and test set, and also performed between primary dataset (GPL24676) and validation dataset (GPL16791).

Module membership (MM): MM(i) = cor (x, ME) is defined to measure the importance of a gene in one module. Gene Significance (GS) is defined as the correlation between the individual genes and the trait. In this study, a gene with MM > 0.8 and GS > 0.2 was defined as candidate gene among modules associated with ALS. The identified WGCNA ALS-related genes were crossed with 763 FIRGs (Supplementary Table 1) to obtain the overlapped FIRGs for further analysis.

### LASSO analysis and validation

Least Absolute Shrinkage and Selection Operator (LASSO) is considered appropriate for high-dimensional data and has strong predictive value and can prevent model overfitting (Tibshirani, 1996). In order to identify ALS from non-neurological controls, the “glmnet” package in R was used to conduct LASSO Regression Analysis (Tibshirani, 1996) on log2 (FPKM + 1) transformed data of overlapped FIRGs. With the LASSO regression analysis, FIRGs with non-zero coefficients were selected as hub genes. A LASSO model (Binomial Lasso) with hub genes expression profiles was incorporated. The risk score (RS) of each sample was calculated using the formula: risk score = Σβi x expression value of genei.

For the validation of LASSO model, the primary cohort from GPL24676 was used for the training set and secondary cohort from GPL16791 were used to validate the model. The pROC package in the R package was used to perform receiver operating characteristic (ROC) curve analysis (Robin et al., 2011).

### KEGG pathway analysis

Amyotrophic lateral sclerosis samples from GSE153960 and GSE174332 were clustered into three groups by K-means clustering using an expression matrix (Shabalin, 2012) of hub FIRGs identified by LASSO analysis. Differentially expressed genes between each ALS group and controls were analyzed with DESeq2. Functional enrichment analysis was done by Enrichr (Chen et al., 2013), *p* < 0.05 was considered as significantly enriched.

### Single-nucleus RNA-seq analysis

Motor cortex neuron single-nucleus RNA-sequencing dataset came from GSE174332, including 17 sporadic ALS cases and 17 pathologically normal controls with similar sex distribution (Pineda et al., 2021). Sporadic ALS is defined as no family history and no defined genetic risk factors (SOD1, C9orf72, TBK1, etc.). The analysis was done with R package Seurat (Hao et al., 2021). Low-quality cells with less than 100 detected genes were removed. DEGs expressed in at least 10% of cells were detected with a fold change of >0.25 (log scale) and tested by non-parameteric Wilcoxon rank sum test.

## Results

### Screening of differentially expressed genes (DEGs)

**FIGURE 1:**
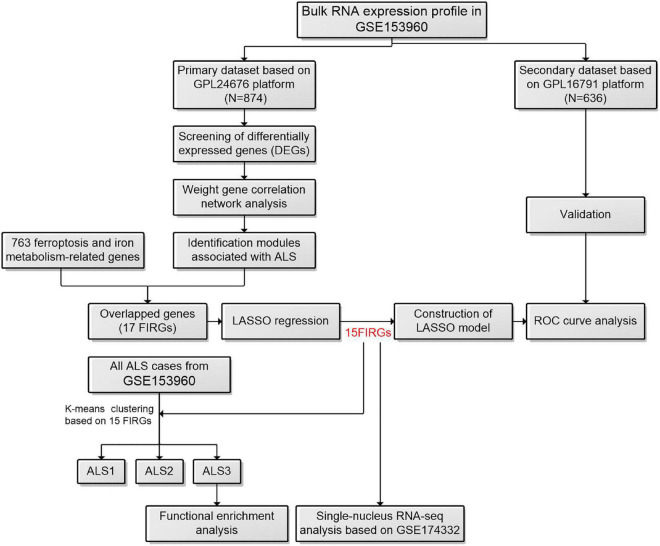
The workflow of this study. ALS, amyotrophic lateral sclerosis; FIRGs, ferroptosis and iron metabolism-related genes; LASSO, least absolute shrinkage and selection operator; ROC, receiver operating characteristic.

**FIGURE 2:**
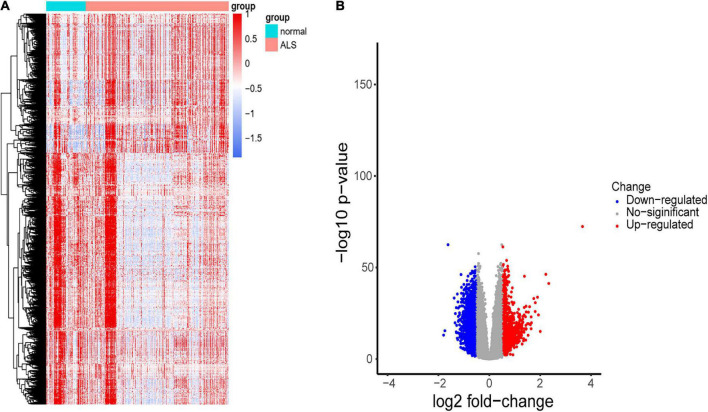
Identification of DEGs between ALS cases and non-neurological controls. **(A)** Heatmap profiling of the genes enrichment analysis between ALS patients and non-neurological controls. **(B)** Volcano plot of DEGs between ALS patients and non-neurological controls. Blue dots represent downregulated genes (1,767 genes), red dots represent upregulated genes (987 genes), and gray dots represent no significantly differentially expressed genes (17,249 genes).

A flowchart of the study is shown in Figure 1. We first investigated the DEGs between ALS patients and non-neurological controls in the primary dataset. As Figure 2 shows, there are 1,767 genes downregulated and 987 genes upregulated in ALS patients, compared with controls. And there are 17,249 genes has no change between ALS patients and controls.

### WGCNA identified critical modules correlating with ALS

**FIGURE 3:**
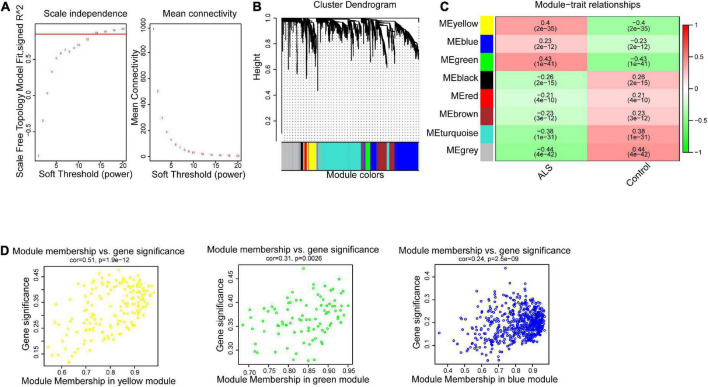
Weighted gene correlation network analysis (WGCNA). **(A)** The scale-free fit index for soft-thresholding powers. The scale-free *R*^2^ > 0.9 was used as a criterion to construct a soft threshold power for appropriate networks. **(B)** A dendrogram of co-expressed gene modules. The branches in the graph are clustered into eight modules, designated by each color below. **(C)** A heatmap shows the association between each module eigengene and phenotype. The coefficient in each cell is the correlation, decreasing from red to green. Yellow, green, and blue three modules are positively correlated with ALS. **(D)** Scatter plot of module eigengenes in the yellow, green and blue modules. All these three modules were significantly associated with ALS (*p* < 0.05).

**FIGURE 4:**
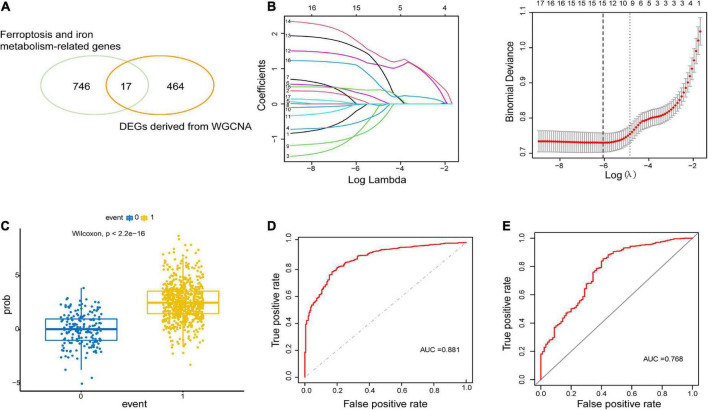
Construction and validation of LASSO model. **(A)** 17 overlapping genes were identified between ferroptosis-related genes and ALS-related DEGs derived from WGCNA. **(B)** Construction of LASSO model. **(C)** Boxplot: visualization of the predictive property of model. Each dot represented each individual’s predicted score. **(D,E)** ROC curve analysis of predicting the occurrence of ALS in the primary dataset and secondary dataset.

To construct the gene co-expression network, WGCNA was used to analyze the expression profiles of DEGs in the primary dataset. On the basis of scale-free *R*^2^ > 0.9, the soft thresholding power was determined as β = 14, and then a scale-free network was constructed (Figure 3A). The module eigengenes were counted and classified into 8 modules designated by a distinctive color (Figure 3B). We explored the correlation of each signature gene with the disease phenotype, as shown in Figure 3C, the yellow (cor = 0.4, *p* = 2e−35), green (cor = 0.43, *p* = 1e−41) and blue (cor = 0.23, *p* = 2e−12) modules were positively related to ALS. The connection between gene significance and module membership for all 3 modules as follows: yellow (cor = 0.51, *p* = 1.9e−12), green (cor = 0.31, *p* = 0.0026) blue: (cor = 0.24, *p* = 2.5e−09) (Figure 3D). To test the stability of the indicated modules, preservation analysis was performed in primary dataset as 1:1 divided into training set and test set (Supplementary Figure 1A), and also performed primary dataset and secondary dataset (Supplementary Figure 1B). Both of the results show green, yellow and blue modules are preserved stably in individual datasets as all of these modules’ Zsummary value of >10 (Supplementary Figures 1A, B). Although blue module’s cor value is smaller than yellow and green modules, its Zsummary value is highest in all modules. There are 860 genes from yellow, green and blue modules. A total of 481 genes that associated with ALS were further identified by MM > 0.8 and GS > 0.2 among three modules. Subsequently, overlap analysis was performed on 481 DEGs identified by WGCNA and 763 FIRGs, and finally 17 overlapped genes were identified (Figure 4A).

### Construction and validation of the LASSO model

The LASSO regression analysis was performed to recognize the optimal linear model of crucial genes for predicting the occurrence of ALS (Figure 4B). A total of 15 FIRGs with non-zero coefficients were selected for model construction. The risk score was calculated as follows:

Risk score=(0.1567×expression value of ANO6)+(0.3361 ×expression value of CD44)+(0.0333×expression value of CHMP5) +(1.3087×expression value of MOSPD1)+(1.6129 ×expression value of NCF2)+(1.8261×expression value of SDCBP)+(0.4153×expression value of STEAP2)+(1.1168 ×expression value of TMEM14C)+(0.0040×expression value of ULK1)-(0.2332×expression value of ACSL4)-(0.8929×expression value of ATP6V0E1)-(0.5481×expression value of B2M)-(0.917×expression value of CYBB)-(0.0261×expression value of CYBRD1)-(0.0096×expression value of HIF1A).

We performed a predictive score based on LASSO analysis, which is helpful in effectively discriminating ALS patients from non-neurological controls (*p* < 2.2e−16, Wilcoxon test) (Figure 4C). Then the ROC curve analysis was implemented on the primary dataset, showing that the area under the ROC curve (AUC) of the model was 0.881 (Figure 4D). Simultaneously, ROC analysis in the secondary validated dataset indicated the AUC value of 0.768 (Figure 4E). These results illustrated that the model based on these 15 hub FIRGs had a valid value in predicting and diagnosing ALS.

### Functional enrichment analysis based on cases that significantly associated with hub FIRGs in ALS

**FIGURE 5:**
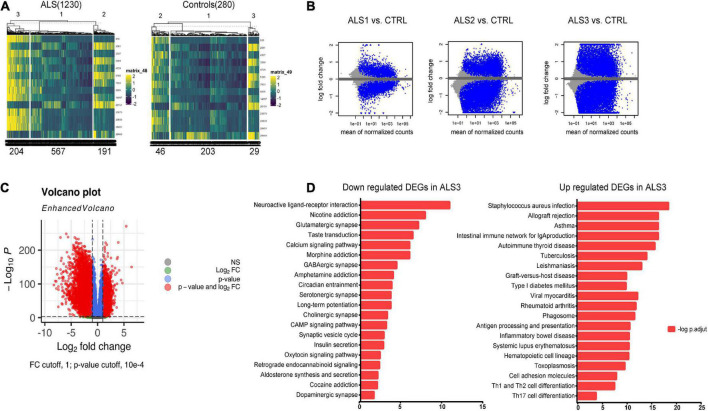
KEGG pathway analysis based on FIRGs that significantly altered in ALS. **(A)** Sample clusters based on expression matrix of 15 hub genes. **(B)** MA plot of DEGs of each ALS subgroup vs. controls. **(C)** Volcano plot of DEGs between ALS3 and controls. **(D)** KEGG pathway analysis of downregulated and upregulated DEGs in ALS3 subgroup, compared with non-neurological controls.

To detect the most significantly influenced function in ALS pathogenesis by 15 hub FIRGs, we clustered all ALS samples from GSE153960 and GSE174332 into three groups by K-means clustering using an expression matrix of 15 FIRGs (Figure 5A). Analysis of DEGs between each ALS group and controls indicated that ALS3 subgroup was the most strongly associated with these 15 FIRGs and closely related with ALS (Figures 5B, C). KEGG pathway analysis of the downregulated genes in ALS3 subgroup mainly enriched in synaptically important pathways involving different synapse types (glutamatergic, serotonergic, cholinergic, and dopaminergic) and known ALS-related pathways like the calcium signaling pathway and cAMP signaling pathway. The upregulated DEGs in ALS3 mainly enriched in phagosome and several immune pathways (Figure 5D).

### Single-nucleus RNA-seq analysis investigate the role of FIRGs in primary motor cortex neurons

**FIGURE 6:**
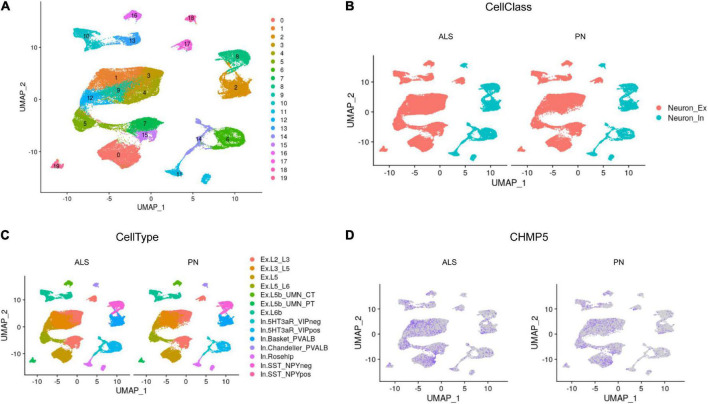
Single-nucleus RNA-seq analysis. **(A)** UMAP plot for the analysis of cell subpopulations in the motor cortex neuron, which were divided into 20 cell subsets. **(B)** The neurons of ALS and PN are classified into excitatory neurons (Neuron_Ex) and inhibitory neurons (Neuron_In), PN: pathologically normal controls. **(C)** Detailed submap of transcriptional isoforms annotated by motor cortex segment excitatory neurons and inhibitory neurons. **(D)** The expression analysis of CHMP5 in motor cortex neuron populations of ALS and PN.

To further investigate the role of FIRGs in primary motor cortex neurons in ALS, this study further extracted primary motor cortex neurons’ single-nucleus RNA-seq data from GSE174332, including 17 sporadic ALS and 17 pathologically normal controls (PN). UMAP analysis done with R package Seurat divided the motor neurons into 20 clusters (Figure 6A). Based on the annotation analysis of cell subpopulations, there are 7 subtypes characterized in the Excitatory neurons (Neuron_Ex) and Inhibitory neurons (Neuron_In), respectively (Figures 6B, C). Thereafter, we studied the expression analysis of the 15 hub FIRGs in cell subpopulations in ALS and PN, and found that CHMP5, one of the 15 hub FIRGs, was expressed significantly higher in excitatory neuron populations of ExL2_L3 and ExL3_L5 in ALS than PN (Figure 6D).

## Discussion

Amyotrophic lateral sclerosis is a multifactorial and highly heterogeneous disease. Although multiple mechanisms have been identified in the pathogenesis of ALS, for example, impaired axonal transport, mitochondrial dysfunction and oxidative stress, immune dysregulation (Mejzini et al., 2019), many mysteries remain in the understanding of ALS pathogenesis. Ferroptosis is a newly identified type of cell death triggered by the accumulation of lipid peroxides, defined as an iron-dependent regulated necrosis that could affect neurons (Stockwell et al., 2017). Previous study raised the hypothesis that the ferroptosis was involved in the pathogenesis of ALS. In SOD1*^G93A^* transgenic mice, reduction of iron level by iron chelators could increase the mean life span (Wang et al., 2011). In ALS patients, increased serum ferritin was positively correlated with a faster progression and shorter survival (Paydarnia et al., 2021).

Here we first investigate the DEGs between ALS patients and non-neurological controls. Using WGCNA, we identify 3 modules DEGs in correlation with ALS. After overlapping with FIRGs, there is a total of 17 FIRGs associated with ALS. Finally, with LASSO analysis, 15 hub FIRGs (ACSL4, ANO6, ATP6V0E1, B2M, CD44, CHMP5, CYBB, CYBRD1, HIF1A, MOSPD1, NCF2, SDCBP, STEAP2, TMEM14C, ULK1) have been screened out as target genes. These hub genes display good predictive value in distinguishing ALS from non-neurological controls, which were validated by secondary dataset.

Ferritinophagy plays an important role in driving some pathological processes in neurodegenerative diseases (Tang et al., 2018). In a lysosomal transport pathway for ferritin, ULK1/2-FIP200 complex regulates ferritin turnover at basal state, and its loss results in TBK1 activation and regulation of ferritin transport (Goodwin et al., 2017). (ULK)1/2 complex, which is also an initial inducer of autophagy. In a recent study of ALS/FTD associated C9orf72, the protein product of C9orf72 could control the initiation of autophagy by regulating ULK1 complex trafficking (Higginbottom, 2016). As a pro-survival factor of ferroptosis, HIF1A is a major transcription factor that can regulate the homeostatic responses to reduced oxygen level in microenvironment (Yang et al., 2019). Moreau et al. (2011) reported that the HIF-1 signaling pathway exhibited significant abnormalities during hypoxia in sporadic ALS cases. The expression of ACSL4 determines sensitivity to ferroptosis. Knockout or inhibition of ACSL4 caused a dramatic reduction in the content of polyunsaturated fatty acids (PUFAs) in membranes, and targeted inhibitors of ACSL4 improved tissue death in a mouse model of ferroptosis (Doll et al., 2017). It is reported that ACSL4 plays important roles in Aβ-induced changes in cell survival, myocardial dysfunction, and lipid peroxidation in AD mice model (Zhu et al., 2022). CYBB (also known as NOX2) participated in the transmission of electrons across the plasma membrane to produce superoxide and other downstream ROS (Bedard and Krause, 2007). In ALS patients, NOX2 activity presented a negative correlation with survival time, while inactivation of NOX2 delayed the progression of neurodegeneration in ALS SOD1 transgenic mice (Sorce et al., 2017). NCF2 is responsible for the synthesis of superoxide in neutrophils. As an oxidative stress gene, NCF2 has been considered as a potential biomarker for Friedreich’s ataxia disease progression and drug action (Hayashi and Cortopassi, 2016). ANO6 is one of the anoctamins, a family of Ca2 + -activated chloride channels and phospholipid scramblases. ANO6 can lead to the activation of lipid peroxidation as a mechanism proposed earlier in ferroptosis (Ousingsawat et al., 2019). In ALS mice model, the ANO6/TMEM16F loss of function reduces denervation and motor decline (Soulard et al., 2020). In contrast, CD44 can inhibit oxidative stress and ferroptosis through regulating GPX4. CD44 can enhance the stability of SLC7A11, which is a protective factor for cells through synthesizes reduced glutathione (GSH) by promoting the uptake of cystine (Liu et al., 2019). Study in SOD1 (G93A) mice showed the expression of CD44 in astrocytes and microglia was accompanied by the pathogenesis of ALS (Matsumoto et al., 2012). CHMP5, a core component of ESCRT-III pathway, has been shown to gather in the plasma membrane during ferroptosis. ESCRT-III could participate in the inversely modulated ferroptosis, mediating plasma membrane repair and decreasing lipid peroxidation. Genetic inhibition of CHMP5 enhanced ferroptosis *in vivo* and *in vitro* (Dai et al., 2020). Previous study showed that higher CHMP5 level in whole blood was positively associated with shorter survival of ALS patients, with potential as a biomarker for ALS prognosis (Zhang et al., 2022). B2M localizes to the cell membrane and is a member of major histocompatibility complex-1 (MHC-1), which participates in synaptic function and axonal regeneration. Widely expressed in human brain, B2M has been shown to play a role in neurodegenerative diseases (Gupta et al., 2017) and has previously been described in sporadic ALS studies (Baciu et al., 2012). SDCBP can interact with a variety of signal proteins, and regulate the different signaling transduction pathways (Pradhan et al., 2020). It is reported that individuals with AD had increased SDCBP expression (Lark and LaRocca, 2022). In addition, CYBRD1, STEAP2 and TMEM14C are significantly involved in iron transport and HEME metabolism. Dysfunction of these genes could cause iron accumulation and iron homeostasis disorders (Ji and Kosman, 2015; Kleven et al., 2015; Harding et al., 2020). However, it’s still unclear about the MOSPD1 and ATP6VE01 involved in the process of ferroptosis, and there is no evidence to support their role in ALS. Overall, these findings may provide a new perspective for exploring the molecular mechanism underlying ALS pathogenesis.

For enrichment analysis, to detect which pathways the FIRGs is mainly involved in the pathogenic process of ALS, we clustered all ALS samples to 3 groups based on the correlation with 15 FIRGs. The group 3 has the most significant correlation with 15 FIRGs. We performed KEGG pathway analysis on all upregulated genes and all downregulated genes in group 3. The result revealed that functional enrichment of downregulated DEGs in ALS 3 is in synaptically important pathways, calcium signaling pathway and cAMP signaling pathway. Progressive synapse loss and dysfunction is known to occur in the early stage of neurodegenerative disease, including Alzheimer’s disease (AD) (Selkoe, 2002), Parkinson’s disease (PD) (Villalba et al., 2015), and ALS (Belzil et al., 2016). Glutamate excitotoxicity, driven by the hyperactivity of excitatory neurons, plays an important role in ALS pathology. One possible reason is the inherent variations in the firing properties of neurons or a reduction in the inhibitory control of GABAergic cells. Interestingly, in KEGG analysis of this study, GABAergic synapse pathway was also enriched of downregulated DEGs in the ALS3 subgroup, suggesting that the inhibitory networks belonged to vulnerability in ALS, consistent with reports in ALS animal models and human cadavers (Maekawa et al., 2004; Allodi et al., 2021; Lin et al., 2021). On the other hand, a recent study revealed that cAMP/PKA activation and neuronal firing were beneficial for synaptic recovery and ameliorated the ALS-related pathobiochemistry (Ba̧czyk et al., 2020). Their hypothesis is that dysfunction of excitatory synapses is an important factor affecting the excitatory drive of motor neurons and the degeneration of motor neurons. Our analysis also found the inactivation of the PKA signaling pathway in ALS3 cases. In addition, the calcium signaling pathway is downregulated in our analysis. Dysregulation of calcium homeostasis participates in the pathogenesis of ALS by affecting some major and interrelated toxicity pathways like oxidative stress, mitochondrial dysfunction and neuroinflammation (Jaiswal, 2013). In the pathways of upregulated DEGs enriched, phagosome plays an important role both in ALS and ferroptosis. Autophagosomes are double-membrane vesicles that participate in the process of autophagy. The main role of autophagy in ALS commonly includes affecting the accumulation of protein aggregates and the function of mitochondria in motor neurons (Taylor et al., 2016). Excessive autophagy, especially selective types of autophagy, and impaired lysosomal activity may drive cells toward ferroptosis. The key types include ferritinophagy, lipophagy, clockophagy, and chaperone-mediated autophagy (CMA) (Liu et al., 2020).

The primary motor cortex is essential for voluntary motor control, sending layer 5 intratelencephalic tract and pyramidal tract output projections to modulate the execution of movement (McColgan et al., 2020). ALS is the most common motor neuron disorder, and show selective vulnerability of motor neurons in cortex (Hammer et al., 1979; Cronin et al., 2007). To further investigate the role of FIRGs in motor cortex neurons in ALS, we further extracted primary motor cortex neurons’ single-nucleus RNA-seq data from GSE174332, which generated the first high-resolution single-cell molecular atlas of the human primary motor cortex. Here we analyzed the 15 FIRGs’ expression in primary motor cortex neurons between 17 sporadic ALS and 17 PN. More interestingly, by analyzing single-nucleus RNA-seq data, we found that in CHMP5, one of the 15 FIRGs, is dysregulated in ExL2_L3 and ExL3_L5, indicating that in ALS patient, excitatory neurons located in motor cortex layer 2_3 and layer 3_5 have higher CHMP5 expression and may be more vulnerable to ferroptosis and iron metabolism dysfunction.

In summary, our results demonstrate that FIRGs are significant factors in the ALS pathogenesis. These pinpointed 15 FIRGs are considered as potential biomarkers for ALS diagnosis. The KEGG analysis shows these FIRGs may involve the ALS pathogenesis by synapse pathways, calcium signaling pathway and cAMP signaling pathway. Finally, single-nucleus analysis reveals higher expression of CHMP5 in L2_3 and L3_5 excitatory neurons in ALS, providing a target gene for further exploration of the pathogenesis and a potential treatment for ALS.

## Conclusion

Taken together, those data indicate that there is a positive correlation between ferroptosis, iron metabolism dysfunction and ALS severity. Thus, these 15 FIRGs are protentional markers of ALS diagnosis and worth further investigation, especially the CHMP5 function in excitatory neurons.

## Data availability statement

The original contributions presented in this study are included in this article/Supplementary material, further inquiries can be directed to the corresponding authors.

## Author contributions

XF analyzed the data and wrote the initial version of the manuscript. YH analyzed a part of the data. ZL and YX designed the study and revised the manuscript. All authors reviewed and approved the final manuscript.

## Footnotes

1
http://zhounan.org/

2
https://www.gsea-msigdb.org/gsea/msigdb/index.jsp

## Supplementary material

Supplementary Figure 1 Module preservation analysis. **(A)** Modules preserved in primary dataset training set vs. test set; **(B)** modules preserved in primary dataset vs. secondary dataset. Each module was represented by its color-code and name. The dashed blue and red lines indicated the thresholds *Z* = 2 and *Z* = 10, respectively. Zsummary < 2 implied no evidence for module preservation, 2 < Zsummary < 10 implies weak to moderate evidence, and Zsummary > 10 implies strong evidence for module preservation.

Supplementary Table 1 The total of 763 FIRGs screened out from the FerrDb database and MsigDB database.
