# Supplementary figures and images for: A conjoint analysis of bulk RNA-seq and single-nucleus RNA-seq for revealing the role of ferroptosis and iron metabolism in ALS
Source: Front Neurosci. 2023 Mar 2;17:1113216. doi: 10.3389/fnins.2023.1113216 (PMC10017473; doi:10.3389/fnins.2023.1113216)

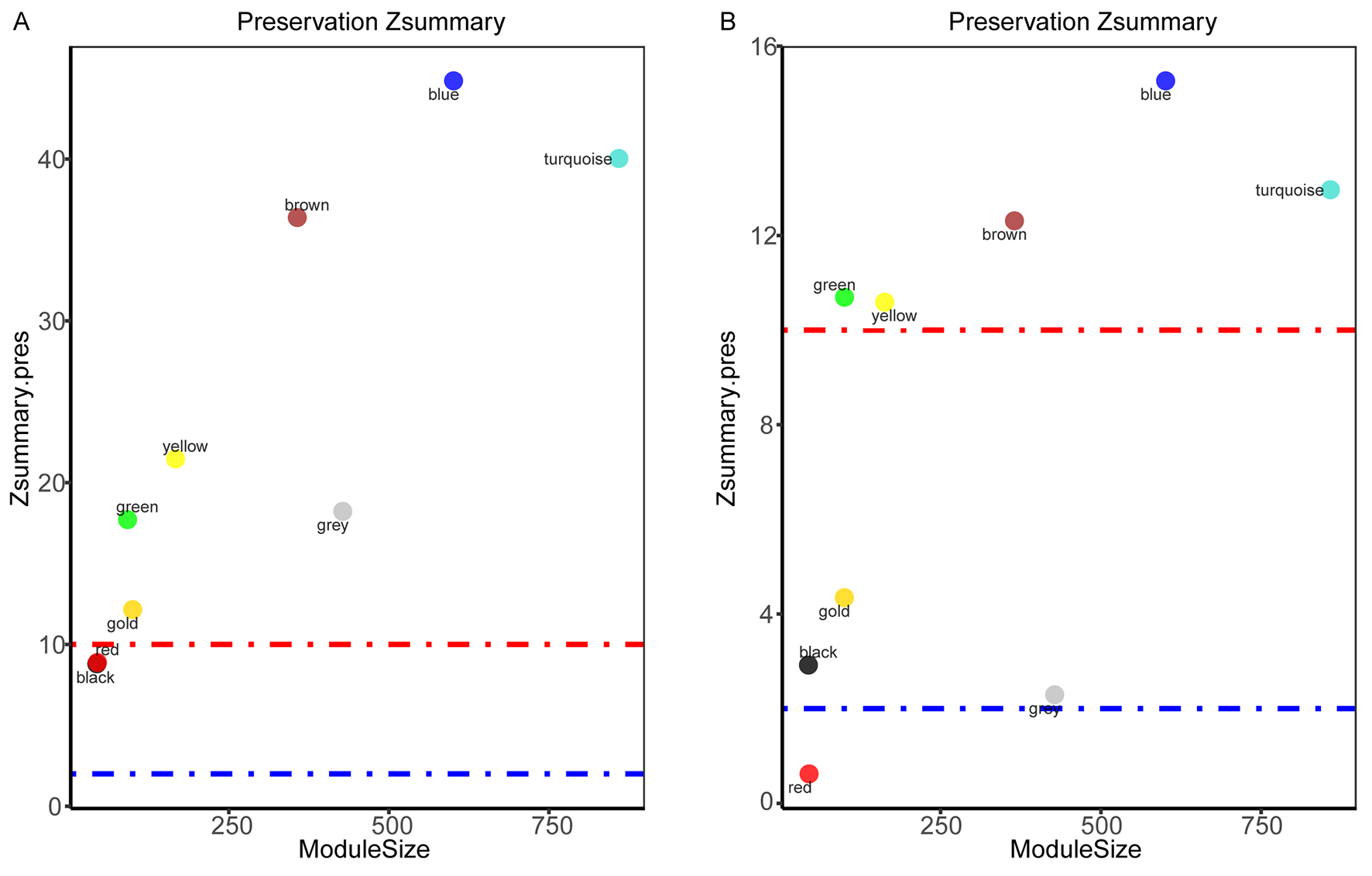

Supplement: Supplementary Figure 1 — Module preservation analysis. (A) Modules preserved in primary dataset training set vs. test set; (B) modules preserved in primary dataset vs. secondary dataset. Each module was represented by its color-code and name. The dashed blue and red lines indicated the thresholds Z = 2 and Z = 10, respectively. Zsummary < 2 implied no evidence for module preservation, 2 < Zsummary < 10 implies weak to moderate evidence, and Zsummary > 10 implies strong evidence for module preservation. [file Image_1.TIF]
